# Induced Pluripotent Stem Cell Derivation and Ex Vivo Gene Correction Using a Mucopolysaccharidosis Type 1 Disease Mouse Model

**DOI:** 10.1155/2019/6978303

**Published:** 2019-04-01

**Authors:** Toshio Miki, Ludivina Vazquez, Lisa Yanuaria, Omar Lopez, Irving M. Garcia, Kazuo Ohashi, Natalie S. Rodriguez

**Affiliations:** ^1^Department of Surgery, Keck School of Medicine, University of Southern California, 2011 Zonal Avenue, HMR 509A, Los Angeles, CA 90033-9141, USA; ^2^Laboratory of Biochemistry and Molecular Biology, Graduate School of Pharmaceutical Sciences, Osaka University, 1-6 Yamadaoka, Suita, Osaka 565-0871, Japan

## Abstract

Mucopolysaccharidosis type 1 (MPS-1), also known as Hurler's disease, is a congenital metabolic disorder caused by a mutation in the alpha-L-iduronidase (IDUA) gene, which results in the loss of lysosomal enzyme function for the degradation of glycosaminoglycans. Here, we demonstrate the proof of concept of ex vivo gene editing therapy using induced pluripotent stem cell (iPSC) and CRISPR/Cas9 technologies with MPS-1 model mouse cell. Disease-affected iPSCs were generated from *Idua* knockout mouse embryonic fibroblasts, which carry a disrupting neomycin-resistant gene cassette (Neo^r^) in exon VI of the Idua gene. Double guide RNAs were used to remove the Neo^r^ sequence, and various lengths of donor templates were used to reconstruct the exon VI sequence. A quantitative PCR-based screening method was used to identify Neo^r^ removal. The sequence restoration without any indel mutation was further confirmed by Sanger sequencing. After induced fibroblast differentiation, the gene-corrected iPSC-derived fibroblasts demonstrated Idua function equivalent to the wild-type iPSC-derived fibroblasts. The Idua-deficient cells were competent to be reprogrammed to iPSCs, and pluripotency was maintained through CRISPR/CAS9-mediated gene correction. These results support the concept of ex vivo gene editing therapy using iPSC and CRISPR/Cas9 technologies for MPS-1 patients.

## 1. Introduction

Recent advancements in gene sequencing technology now allow us to access genetic information of patients in order to design personalized therapies [1, 2]. Especially in the case of monogenic diseases, as are most of the congenital metabolic disorders (CMDs), identification of the genetic mutation underlying the disease is essential not only for precise diagnosis but also for the identification of potential target sequences to apply genome editing therapeutic strategies. The genome editing approach is aimed at directly modifying or correcting the disease-associated mutation in the patient's genome and is of great interest in the prevention and treatment of a number of genetic diseases [3].

Several molecular tools that introduce DNA double-strand breaks (DSBs) at specific sites have been developed and utilized in biomedical research [4]. Of these, a clustered regularly interspaced short palindromic repeat-associated 9 (CRISPR/Cas9) system, which is able to achieve highly flexible and specific targeting, has superior advantages and represents a powerful tool for potential clinical application. The CRISPR/Cas9 technology derives from the adaptive immunity evolved in bacteria such as *Streptococcus pyogenes* to defend against invading plasmids and viruses [5]. After binding to a conserved sequence named protospacer-adjacent motif (PAM), Cas9 generates a blunt DSB. The DSB subsequently is repaired by either nonhomologous end joining (NHEJ) or homology-directed repair (HDR). During this DSB repair process, pathogenic mutations can be replaced with a donor DNA homologous template containing a corrected gene sequence [4].

One potential use for this powerful gene editing tool in the treatment of CMDs is the ex vivo gene correction approach, which is aimed at correcting mutations in the patient's cells outside the body. Because this approach requires efficiently proliferating cells; it has been proposed to combine *ex vivo* gene correction with stem cell technologies already in place [3]. Somatic cells isolated from CMD patients can be reprogrammed to induced pluripotent stem cells (iPSCs) by overexpressing specific transcription factors [6]. These iPSCs possess pluripotency, the ability to differentiate into all three germ cell layers, and unlimited replicative potential. Following gene correction, these patient-specific iPSCs could be differentiated into the desired cell type and transplanted back into the patient [7].

Here, we focus on one of the CMDs, mucopolysaccharidosis type 1 (MPS-1). MPS-1 is an autosomal recessive disorder caused by a mutation of a gene that is responsible for the expression of the *α*-L-iduronidase (IDUA) enzyme [8]. The lysosomal enzyme, IDUA, is involved in the breakdown of sulfated glycosaminoglycans (GAGs) such as heparan sulfate and dermatan sulfate [8]. The lack or shortage of the IDUA enzyme leads to the accumulation of GAGs in the lysosomes of all organ systems. MPS-1 has different clinical representations based on the severity of the disease, including developmental delay, coarsened facial features, skeletal and joint abnormalities, airway obstruction, corneal clouding, and hepatosplenomegaly. MPS-1 severely affects the patient's quality of life and can be lethal within the first decade of life if left untreated. Current treatments for MPS-1, including enzyme replacement therapy and bone marrow transplantation, are symptomatic therapies, and novel definitive treatments are needed [9].

**Figure 1:**
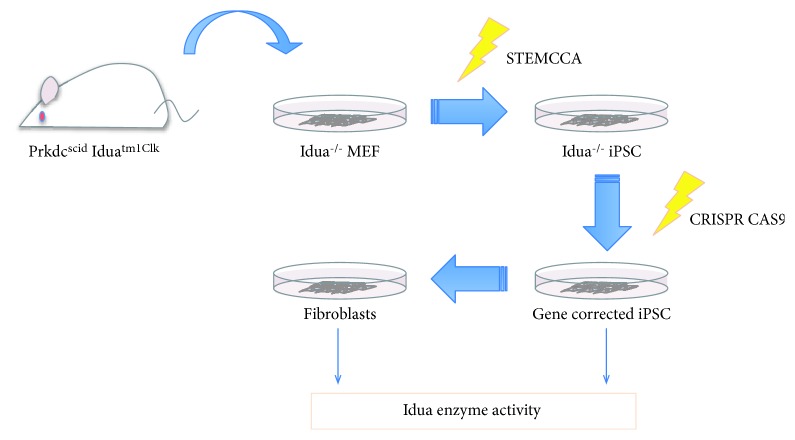
Schematic representation of the Idua^−/−^ iPSC derivation and gene correction. Idua^−/−^ mouse embryonic fibroblasts were reprogrammed with STEMCCA lentivirus. The disrupting neomycin resistance gene (Neo^r^) was removed, and the Idua gene was corrected with CRISPR/Cas9 gene editing technology.

In the present study, we demonstrate the proof of concept of the autologous stem cell-based ex vivo gene correction approach for the treatment of MPS-1 by using the disease model animal, Idua knockout (KO) mice [10]. First, iPSCs were derived from the Idua KO mouse embryonic fibroblasts, in which exon VI of the IDUA gene was disrupted by a neomycin-resistant gene cassette (Neo^r^). Then, we removed the interruption construct (Neo^r^) by CRISPR gene editing [11]. The restoration of Idua enzyme function was validated in the gene-corrected mouse iPSC and in fibroblasts that were differentiated from the gene-corrected miPSCs (Figure 1).

## 2. Materials and Methods

### 2.1. Mice

All mice used in this study were bred and euthanized appropriately following the protocols that were approved by the University of Southern California Institutional Animal Care and Use Committee and conducted following the NIH Guide for the Care and Use of Laboratory Animals. Breeder heterozygous pairs of NOD.129 (B6)-Prkdc^scid^ Idua^tm1Clk^ mice were obtained from The Jackson Laboratory (#004083), housed under specific pathogen-free conditions and provided with regular chow (TEKLAD #2018) and sterile/acidified water. PCR-based genotyping was performed with specific primers according to The Jackson Laboratory's instructions.

### 2.2. Mouse iPSC Derivation and Maintenance

Mouse embryonic fibroblasts (MEFs) were isolated from individual 13.5 d.p.c embryos, as described previously, with some modifications [6]. The passage 4 MEFs were used for iPSC generation. Reprogramming was conducted with a single lentiviral vector, STEMCCA [12], kindly provided by Dr. Gustavo Mostoslavsky (Boston University), which contains OCT4, SOX2, KLF4, and c-MYC. Approximately 100,000 MEFs were infected with lentiviral particles containing STEMCCA in 1 mL of basal mouse embryonic stem (ES) cell medium (DMEM supplemented with 10% FBS, 2 mM GlutaMAX, 0.1 mM nonessential amino acids, 1 mM sodium pyruvate, 0.1 mM *β*-mercaptoethanol, and 50 units/50 mg/mL penicillin-streptomycin) with 6 *μ*g/mL polybrene (All from Gibco/Invitrogen). A second transduction was performed with the same conditions after 24 h. On the following day, cells were dissociated with brief trypsinization and reseeded onto an irradiated MEF feeder layer. The culture medium was changed to 1,000 U/mL leukemia inhibitory factor (LIF, Chemicon) containing mouse ES culture medium on the next day and changed every other day thereafter. Colony formations were examined daily by microscopic observations. After derivation, all iPSCs were maintained in feeder-free 2i/LIF conditions which contained 1,000 U/mL leukemia inhibitory factor (LIF, Chemicon) and 1 *μ*M PD032901 and 3 *μ*M CHIR99021 on Matrigel-coated plates. Three cell lines (L13, L14, and L15) were chosen according to the morphology of the colonies and used for this study. Mouse ES cell line (D3) was also cultured under 2i/LIF conditions and used as positive control.

### 2.3. Pluripotent Stem Cell Marker Immunofluorescent Staining

Pluripotent stem cell characteristics of the three Idua^−/−^ miPSCs were validated with immunofluorescent staining for stem cell marker proteins. Cells were plated on 8-well chamber slides coated with Matrigel until colonies formed. Slides were then fixed with 4% paraformaldehyde, permeabilized with 0.3% Triton-X, and incubated with 5% Goat Serum. Antibodies for SSEA-1 (MC480 mAB, Thermo Fisher Scientific), Oct4a (C30A3 rabbit mAB, Cell Signaling Technology), and anti-mouse/anti-goat IgG/IgM isotype control (BD Pharmigen) were used as primary antibodies. Alexa Fluor 594 goat-anti-mouse IgG and Alexa Fluor 488 goat-anti-rabbit IgG were used accordingly as secondary antibodies. ProLong® Gold Antifade Reagent with DAPI (Life Technologies) was used for nuclear counter staining and mounting with coverslips.

### 2.4. Stem Cell Gene Expression Validation

Total RNA was isolated using a Direct-zol RNA Mini Prep Kit (Zymo) following the manufacturer's protocol, and the cDNA was synthesized from 1 *μ*g RNA using qScript™ cDNA SuperMix (Quanta Biosciences). Quantitative real-time PCR (qRT-PCR) was carried out using the ViiA™ 7 Real-Time PCR System (Life Technologies) with TaqMan Gene Expression Master Mix (Applied Biosystems) and the TaqMan Gene Expression Assay primers for Oct 4, Sox2, Nanog, and GAPDH. All reactions were carried out on the same plate in duplicate.

### 2.5. Induced Spontaneous Cell Differentiation

Idua^−/−^ miPSC lines, L13, L14, and L15, and wild-type control miPSC lines were trypsinized into a single cell suspension and plated onto a low attachment 6-well culture plate with basal mES media (without LIF) for 3 days to induce embryoid body (EB) formation. Further spontaneous differentiation was induced by culturing EBs by supplementing retinoic acid (100 *μ*M) or 0.1% dimethyl sulfoxide. Each culture condition was reproduced in the 4-well chamber slides for immunofluorescent analyses. Antibodies for b3-tubulin (MA1-118, Invitrogen), *α*-actinin (AT6/172, abcam), *α*-fetoprotein (F1-6P2A8-P2B9A9, Invitrogen), and anti-mouse/anti-goat IgG/IgM isotype control (BD Pharmigen) were used as primary antibodies.

### 2.6. Alpha-L-Iduronidase (IDUA) Activity Assay

The intracellular IDUA activity in the three Idua^−/−^ miPSC and iPSC-derived fibroblasts were evaluated with an established *in vitro* activity assay [13]. Sodium formate, formic acid, 4-methylumbelliferone, glycine, sodium hydroxide, Triton X-100, and sodium azide were obtained from Sigma-Aldrich (St. Louis, MO) and 4-methylumbelliferyl alpha-L-iduronide from Glycosynth (Warrington, Cheshire, UK). Briefly, cells were homogenized by ultrasonic sonication (QSonica Q700, Newtown, CT) in 100 *μ*L cold PBS over ice at a pulse mode rate of 10 seconds, three times, at 40% amplitude and set temperature of 4°C. Homogenate was centrifuged for 10 minutes at 4°C, 13,000 rpm to recover cytosolic protein IDUA enzyme. 10% Triton X-100 in PBS was added, and the homogenate (tissue/cell) was kept on ice for 10 min. The equal volume of a solution of 50 *μ*M 4-methylumbelliferyl alpha-L-iduronide and the tissue homogenate were mixed and incubated for 1 h at 37°C in the dark. The reaction was quenched by adding 0.5 M NaOH/glycine buffer. Tubes were centrifuged for 1 min at 13,000 rpm at 4°C, the supernatant was transferred to a 96-well plate, and fluorescence was read at 365 nm excitation wavelength and 450 nm emission wavelength using a SpectraMax M5 fluorometric plate reader (Molecular Devices, Sunnyvale, CA). The specific activity of IDUA was expressed as nmol/h/mg protein in each sample.

### 2.7. CRISPR Gene Editing

The GeneArt CRISPR Nuclease (OFP Reporter) Vector Kit (Thermo Fisher Scientific, Cat#. A21174) was used for this project. The crRNAs were designed by the CRISPR Design MIT website. Briefly, the designed single-stranded oligonucleotides were annealed and cloned into the CRISPR Nuclease vector by using T4 DNA ligase. Two vectors and donor template oligonucleotides were transfected to about 500,000 miPSCs with Lipofectamine 3000 (Thermo Fisher Scientific, Cat#. L3000015). After 3 days, the orange fluorescent protein (OFP, Ex548/Em560) expressing cells were sorted by a MoFLo Astrios Cell Sorter (BECKMAN COULTER). The OFP-positive miPSCs were plated at low density until small colonies were visible. miPSC-like colonies were transferred to a 96-well plate. The 96-well plate was later duplicated. One plate was used for DNA extraction, and another plate was used to cryopreserve the cells. Cells were cryopreserved in 10% DMSO containing ES culture grade FBS and stored in cryopreserve tubes until the screening for gene editing was completed. Screening primers were designed with Primer 3 software. DNA was extracted from 96-well plates using a 96-well genomic DNA extraction kit (Zymo) and sequenced by Laragen Sequencing and Genotyping Company. CRISPR/Cas9-mediated double-stranded cleavage efficiency was detected using a mismatch cleavage assay using the GeneART® Genomic Cleavage Detection Kit (Cat#. A24372).

### 2.8. Teratoma Formation Assay

Teratoma formation was examined by transplanting cell under the kidney capsule space as previously described [14, 15]. In brief, miPSCs were resuspended with serum-free DMEM with an equal volume of EHS-gel (Matrigel; BD Biosciences, Bedford, MA, USA) to a final ratio of 1.5 × 10^6^ cells/100 *μ*L. A total of 1.5 × 10^6^ miPSCs were transplanted under the left kidney capsule space. The recipient mice were anesthetized by isoflurane inhalation. The teratoma samples were resected and fixed with 4% (*v*/*v*) phosphate-buffered formalin, and paraffin-embedded sections were stained using with hematoxylin and eosin (H&E staining) according to standard procedures.

### 2.9. Fibroblast Differentiation

To test the restoration of Idua activity, 2 lines of gene corrected iPSC, Idua-KO iPSCs, and wild-type iPSCs were differentiated by using modified fibroblast differentiation protocol [16]. Briefly, cells were cultured with DMEM, 5% FBS, 0.5 *μ*g/mL hydrocortisone, 10 ng/mL EGF, and 1% Insulin-Transferrin-Selenium on 0.1% gelatin-coated dishes for 7 days. Cells were passaged onto uncoated plates and harvested on day 21 to test the Idua activity.

### 2.10. Statistical Analysis

Results are expressed as the mean ± standard error of the mean (SEM). Statistical analysis was performed with Prism 5.0a (GraphPad Software, San Diego, CA, USA). Experimental and control groups were compared with paired or unpaired one-way ANOVA (with Bonferroni post hoc analysis and Dunnett's multiple comparison). A value of *P* < 0.05 was considered statistically significant.

## 3. Results

### 3.1. Generation and Characterization of MPS-I Disease-Specific Mouse iPSC

**Figure 2:**
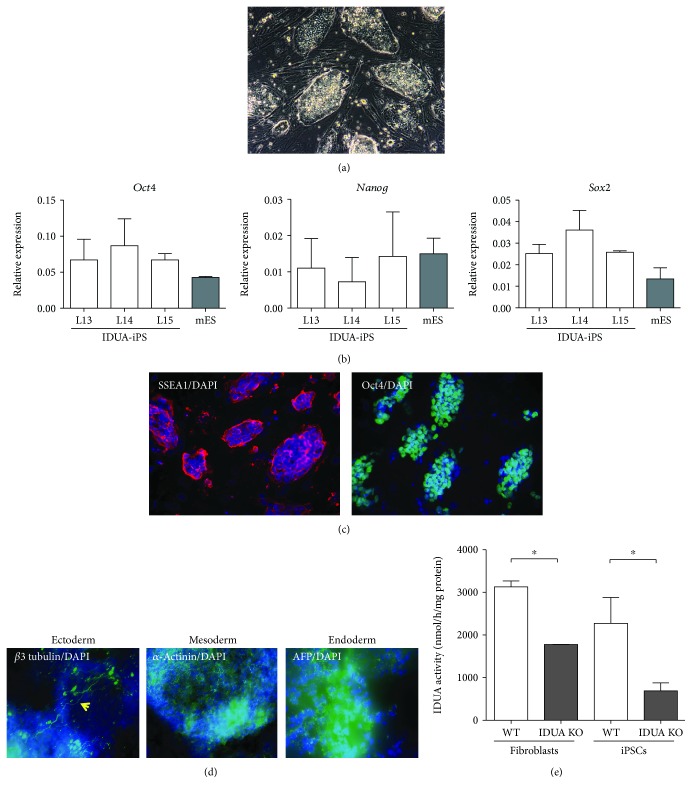
Idua^−/−^ mouse iPSC derivation and validation. (a) Phase contrast image of miPSC colonies. (b) Stem cell marker gene expression in three different miPSC lines. Mouse embryonic stem cell (mES) as control. (c) Immunofluorescent staining for stem cell markers, SSEA-1 and Oct4. Nuclear counter staining with DAPI. (d) Immunofluorescent staining demonstrated lineage-specific marker protein-positive cells. (e) Biochemical analyses demonstrate IDUA activity in wild-type (WT) and IDUA knockout (IDUA KO) fibroblasts and iPSCs derived from each cell line.

Mouse fibroblasts isolated from ED13.5 Idua^−/−^ mouse embryos were used to induce pluripotency with STEMCCA lentivirus [12]. Reprogramming efficiency was sufficiently high to obtain over 20 colonies from 10^5^ MEFs (Figure 2(a)). Of these, 3 iPSC lines were selected based on the morphology and used for the following validation studies. Wild-type sibling MEFs were also reprogrammed in the same manner to generate wild-type mouse iPSCs as the control.

All three iPSC lines showed stem cell marker transcription factors, *Pou5f1* (*Oct4*), *Sox2*, and *Nanog* mRNA expression equivalent to that of mouse embryonic stem cells (Figure 2(b)). Immunofluorescence staining demonstrated mouse ES cell surface marker SSEA-1 and transcription marker Oct4 expression at the cell surface and nuclear region, respectively (Figure 2(c)).

The pluripotency of these miPSCs was examined by in vitro differentiation induction. All three germ layers, ectoderm, mesoderm, and endoderm differentiations, were induced with retinoic acid or dimethyl sulfoxide supplementation into the culture media. Differentiation to *β*3-tublin-positive neuronal cells (ectoderm), *α*-actinin-positive cardiac cells (mesoderm), and *α*-fetoprotein-positive cells (endoderm) was confirmed with immunofluorescence staining (Figure 2(d)). These immunofluorescence staining data confirmed that all three Idua^−/−^ MEF-derived iPSC lines acquired pluripotency by lentiviral overexpression of four reprogramming factors. Regarding the differentiation capability (frequency of the marker protein-positive cells), there were no noticeable differences between the Idua^−/−^ miPSCs and control wild-type miPSCs. These data indicate that the lack of functional Idua did not affect iPSC derivation.

We further confirmed that the Idua^−/−^ MEF-derived iPSC carried the disease trait by measuring the Idua enzyme activity. The Idua activity was significantly lowered (*P* < 0.005) in the Idua^−/−^ miPSCs compared to that of wild-type miPSCs as well as the original fibroblasts (Figure 2(e)).

### 3.2. CRISPR Gene Editing Approach and Efficiency

**Figure 3:**
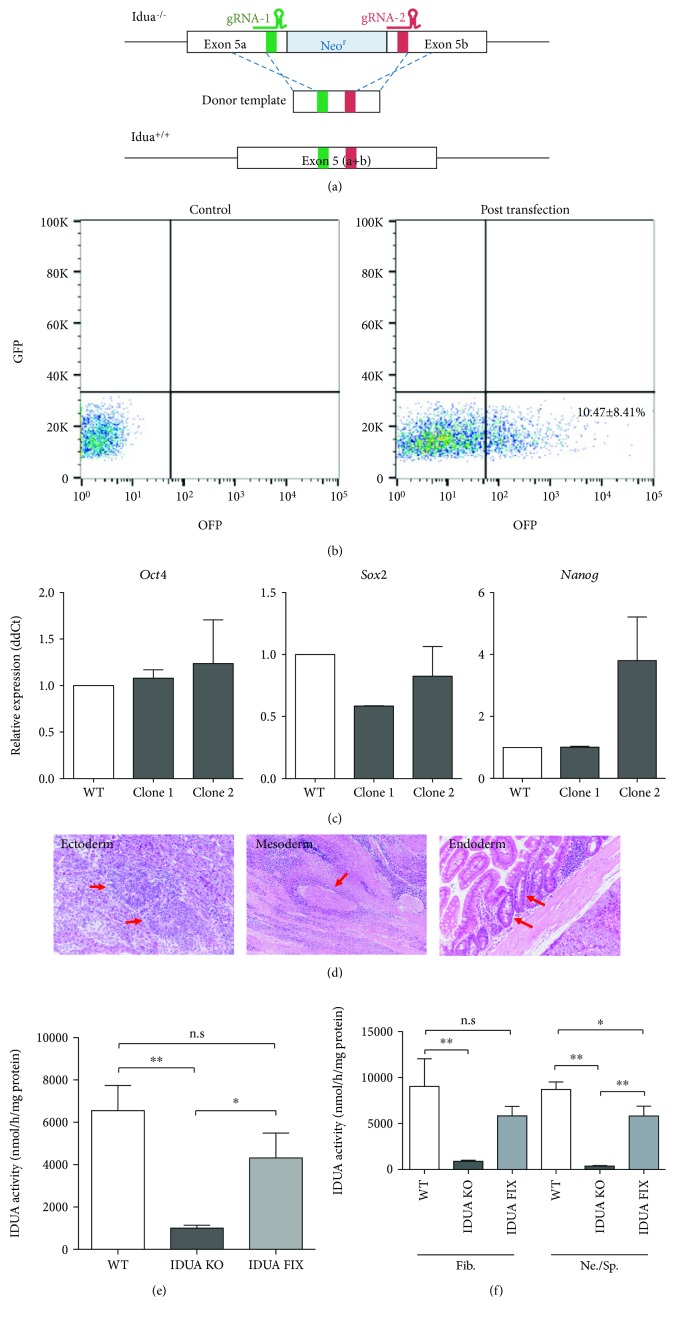
CRISPR gene editing and validations. (a) Schematic representation of CRISPR gene editing strategy. (b) Flow cytometric analysis of CRISPR/Cas9-OFP vector transfection efficiency. GFP channel was used to detect autofluorescence of dead cells. (c) Stem cell marker gene expression in two gene-edited miPSC lines. (d) Identification of three germ layers in teratomas. Red arrows indicate typical histological feature of each lineage. (e) Biochemical analyses to validate Idua enzyme function restoration. ^∗^ *P* < 0.05 and ^∗∗^ *P* < 0.005. n.s: no significant difference using one-way ANOVA with Bonferroni post hoc analysis and Dunnett's multiple comparison. (f) The retention of restored Idua enzyme activity was confirmed after inducing cell differentiation (Fib.: fibroblast-like cells, Ne./Sp.: spontaneously induced neuronal-shape cells). ^∗^ *P* < 0.05 and ^∗∗^ *P* < 0.005. n.s: no significant difference using one-way ANOVA with Bonferroni post hoc analysis and Dunnett's multiple comparison.

The Idua^−/−^ transgenic mice were generated by inserting a neomycin resistance gene (Neo^r^) into the exon VI of the Idua gene [10]. First, we characterized the genomic region within exon VI of Idua gene and designed pairs of gRNAs from the sequence before and after the inserted Neo^r^ gene (Figure 3(a)). Based on the sequencing data, donor templates and different sets of crRNA inserts were designed. Mouse genomic Idua gene sequence (NC_000071.6) from the NCBI database was used as the reference sequence. Three different homology arm size donor templates were designed: 97 bp, 213 bp, and 543 bp (Supplementary Information 2). Of these, 97 bp successfully reconstructed the Idua gene. The canonical PAM is the sequence 5′-NGG-3′. To prevent recleavage by the Cas9 enzyme after the successful gene correction, one of the two guanine (“G”) nucleobases of PAM recognition site was replaced to adenine (“A”), which code the same amino acid (Supplementary Information 2 and 3). A total of 6 crRNAs was designed and tested the cleavage efficiency with Idua^−/−^ MEF (Supplementary Information 1). Of those, we selected the most efficient pair of crRNA (#1 and #2) for the following miPSC gene editing.

Two CRISPR vectors and donor template oligos were transfected by a lipofection method. After 3 days in culture, the marker protein, orange fluorescent protein (OFP), expressing cells was isolated by fluorescence-activated cell sorting. The average transfection rate was 10.47 ± 8.41% (*n* = 6), and on average, 116,415 cells were sorted from each sample (Figure 3(b)). Sorted cells were then seeded at low density and allowed to form minuscule individual colonies. Each colony was picked up and transferred to a 96-well plate. Genomic DNA was isolated from a total of 149 colonies and screened by PCR with primers specific for Neo^r^ and Idua genes. The samples negative for Neo^r^ gene and positive for Idua with appropriate amplicon size were sequenced by regular Sanger sequencing. Among them, 4 colonies successfully confirmed the collected Idua sequence without insertion and deletion (In/Del). However, only 2 of them could recover as undifferentiated miPSCs.

The two Idua-corrected miPSC clones were further investigated for their pluripotency and Idua activity.

### 3.3. Pluripotency Validation of Gene-Edited miPSC

To verify that the genetically corrected cells retained their pluripotency, *Pou5f1 (Oct4)*, *Sox2*, and *Nanog* mRNA expressions were measured by qRT-PCR (Figure 3(c)). Both established miPSC clones showed this stem cell marker transcription factor mRNA expression was equivalent to that of mouse embryonic stem cells. The pluripotency of the two Idua-corrected miPSC clones was also confirmed by in vivo teratoma formation assay. The miPSC clones were cryopreserved over 6 months with a cryopreservation medium, STEM-CELLBANKER (amsbio). Both clones formed tumor masses under the kidney capsule of Idua KO mice after 7 weeks. The masses contained various histological components of the three germ layers. The tumors partly showed neural rosette-like structures (ectoderm), cartilage-like structures (mesoderm), or gut-like epithelium (endoderm) (Figure 3(d)). These findings indicated that both Idua-corrected miPSC clones maintained their in vivo pluripotency after the CRISPR gene editing, over 10 passages and cryopreservation processes.

### 3.4. Restoration of Idua Enzyme Function

The restoration of Idua enzyme function was confirmed with the biochemical Idua assay (Figure 3(e)). Idua enzyme activity was significantly restored after the CRISPR gene editing process (*P* = 0.0209). The mean value of Idua activity was increased more than 4 times, from 1004 ± 242.7 nmol/h/mg protein to 4319 ± 1175 nmol/h/mg protein. There was no significant difference of Idua activity between the wild-type miPSC and the gene-corrected miPSC (*P* = 0.2495).

In order to confirm the retention of restored enzyme function after cell differentiation, the Idua activity was further confirmed after spontaneous and fibroblast differentiation of the CRISPR-corrected iPSCs (Figure S2A-C). Despite the different differentiation protocols used, both iPSC-derived fibroblast-like cells (Fib: 5837 ± 1022 nmol/h/mg) and spontaneously induced neuronal shape cells (Ne/Sp: 5824 ± 1066 nmol/h/mg) showed equivalent IDUA enzyme activity (Figure 3(f)). As observed in the comparison of undifferentiated iPSCs (Figure 3(e)), the IDUA-corrected iPSC-derived cells maintained significant restoration of IDUA activity. The iPSC-derived fibroblast-like cells showed 6.42 times higher IDUA activity (*P* = 0.0049) whereas spontaneously induced neuronal shape cells showed 14.86 times higher IDUA activity (*P* = 0.0038) compared to the Idua-KO iPSC-derived cells. These findings indicated that the CRISPR-corrected IDUA activity is retained after spontaneous and guided differentiation induction.

## 4. Discussion

This study demonstrates the feasibility of induced pluripotent stem cell (iPSCs) derivation from alpha-L-iduronidase deficient cells and targeted gene correction in these iPSCs using CRISPR/Cas9 technology. We reprogrammed mouse embryonic fibroblasts with all Yamanaka reprogramming factor genes, Oct4, Sox2, Klf4, and cMyc, by using a single cassette lentiviral vector. Since this reprogramming technology was first reported, much effort has been devoted to developing clinically applicable iPSC derivation methods. Patient somatic cells can be isolated from the patient's skin, blood, and even urine for reprogramming. Integration-free reprogramming methods have been developed with transient RNA or protein transfections. These clinically applicable reprogramming methods can be applied to generate therapeutic, patient-specific iPSCs. Our data indicate that one of the lysosomal enzymes, alpha-L-iduronidase, is dispensable in the reprogramming of cells. The lack of Idua enzyme did not limit iPSC colony expansion or cryopreservation. Further detailed studies are required to investigate the long-term impact of Idua deficiency. However, our finding is encouraging and important for the future applications of iPSC technology in basic research and clinical therapy development for MPS-1 patients.

It has been shown that reprogramming also resets telomere length and epigenetic markers [17]. Therefore, it is believed that iPSCs can be maintained and clonally expanded, unlike primary cells. These advantages support the combination of the ex vivo gene correction approach with iPSC derivation technology. The unlimited clonal expandability allows the cells to undergo the *in vitro* gene editing process. After the aimed gene correction, the iPSCs can be differentiated into the desired type of cell and transplanted back into the patient. Since the immunotype would be maintained, this cell transplantation may not require life-long immunosuppression.

Through the process of CRISPR genome editing, from transfection to gene-corrected iPSC colony establishment, the efficiency was lower than expected. There are three unpredictable steps that affect efficiency: transfection, double strand cleavage, and template insertion. After lipofection, the OFP signal indicated the transfection rate, which was about 10% (Figure 3(b)). Practically, a fluorescent marker or antibiotic resistance gene containing selection system can be applied to assess transfection efficiency. Our preliminary sequencing data showed 89% OFP-positive cells possessed in/del mutations at the targeted sequences, which indicated that the efficiency of double-strand cleavage was relatively high. However, the qPCR-based screening indicated only 2.68% of the cells had successfully restored the Idua gene. This low efficiency of correct template insertion might be due to the size of the targeted Neo^r^ sequence. The Neo^r^ cassette used in this study is 795 bp, which might not be ideal to reconstruct apart from the double strand cuts. Most cases of mucopolysaccharidosis (MPS) type I disease involve a single mutation. Therefore, in clinical application, the target region will be much smaller than the Neo^r^ cassette. If the gap size is the reason for the low efficiency of gene editing, we can expect that clinical gene correction efficiency will thus be better than that achieved in this study.

In addition to these molecular biological efficiencies, the cell biological factors also affected the establishment of gene-corrected iPSC colonies. Since the single-clone colonies were picked and placed in individual wells, this may have contributed to their low survival, as they prefer to grow in a semiconfluent environment. It is known that human iPSCs are more sensitive than mouse iPSCs and require higher skill to culture and for maintenance of the colonies. The gene editing process and the following clonal expansion process might decrease the efficiency in establishing patient-specific gene-corrected iPSCs. These processes might also cause undesired differentiation in the iPSC. Indeed, we lost 2 iPSC lines (50%) during the recovery process from cryopreservation. However, as long as the gene correction is established, the pluripotency could be induced by repeating the reprogramming process.

There are two possible genome editing approaches to treat monogenic diseases: ex vivo and *in vivo* gene corrections. The *in vivo* gene correction approach is aimed at inserting a functional enzyme gene or correcting the mutations directly *in vivo* by delivering gene editing tools to the desired genomic site in the specific tissue. Sharma et al. demonstrated the proof of concept of the *in vivo* gene editing approach for lysosomal storage disorders [18]. Zinc finger nuclease (ZFN) gene editing technology was applied by using an adenoassociated viral (AAV) vector to insert human IDUA gene in the mouse albumin locus. One of the concerns with this approach is that the control of gene expression depends on albumin promoter activity. To achieve more physiological enzyme expression, repairing the patient's own gene sequence as in the proper chromosome may be more suitable. As applying the concept demonstrated here, transfecting Cas9 protein, two guide RNAs, and a corrected gene template with an AAV vector would be an ideal approach to treat monogenic diseases such as MPS-1.

Overall, the current work remains to confirm iPSC derivation from Idua-deficient cells and *in vitro* IDUA gene correction using CRISPR/Cas9 technology. Although this study demonstrates a feasibility of the promising concept to treat MPS-1 patients, the approach is still experimental and there are a number of hurdles that need to be overcome. The most important issue is safety. This approach utilizes two cutting edge biotechnologies, iPSC and CRISPR, of which safeties are not established yet. Tumorigenicity associated with the iPSC derivation and CRISPR-mediated off-target gene editing remain a concern for clinical application. There are two potential mechanisms that cause tumorigenicity of iPSCs. One is associated with the reprogramming process. Fortunately, efforts of finding safer reprogramming factors and vectors have successfully decreased the risk associated with the reprogramming process [19, 20]. The contamination of remaining undifferentiated cells in iPSC-derived therapeutic cells is the other potential risk. Many approaches have already been proposed to distinguish these undifferentiated cells to decrease the risk of tumorigenicity [21, 22]. However, there are still limitations to apply these safety methods to large numbers of iPSC-derived cells. Among the available gene editing technologies, the CRISPR/Cas9 technology is considered the most precise gene editing tool. The guide RNA-mediated gene targeting mechanism is well studied and believed to have a minimal chance of off-target gene modification effects. However, the frequency of CRISPR-mediated off-target effects is still under debate as of March 2018 [23]. Comprehensive high-resolution sequencing and karyotyping will be required to validate clinically applicable CRISPR gene-edited iPSCs going forward. Further large-scale studies with the latest CRISPR technologies will also move this powerful gene editing tool toward clinical application.

## 5. Conclusions

We have shown that iPSC derivation and CRISPR gene correction are feasible with cells from a lysosomal storage disorder rodent model, MPS-1 Hurler's disease. Considering the unlimited expandability of iPSCs and physiological gene expression after genome editing, this approach is suitable to investigate for therapeutic potential in monogenic inherited metabolic diseases. Further technology advancement and large-scale preclinical evaluation will be required to improve our understanding of the CRISPR genome editing technology and for the future analysis of novel therapeutic interventions.

## Data Availability

The primers, guide RNAs, and donor template sequence data used to support the findings of this study are included within the supplementary information file.

## Abbreviations

IDUA: Alpha-L-iduronidase
ES: Embryonic stem
iPS: Induced pluripotent stem
MEF: Mouse embryonic fibroblasts
CRISPR: Clustered regularly interspaced short palindromic repeats
SSEA: Stage-specific embryonic antigen
Oct-4: Octamer-4
Sox-2: Sex-determining region Y-box 2
DMSO: Dimethyl sulfoxide
OFP: Orange fluorescent protein.

## Supplementary Materials

Supplementary Materials Figure S1: (A) schematic representation of donor template designing strategy. (B) Computer-generated sequencing electropherogram of Idua gene-corrected iPSC. Figure S2: phase contrast images of gene-corrected miPSCs: (A) before inducing differentiations; (B) 21 days after spontaneous differentiation induction; (C) 21 days after fibroblast differentiation induction. Scale bars: 100 *μ*m.
