# Supplementary material for: Induced Pluripotent Stem Cell Derivation and Ex Vivo Gene Correction Using a Mucopolysaccharidosis Type 1 Disease Mouse Model
Source: Stem Cells Int. 2019 Apr 1;2019:6978303. doi: 10.1155/2019/6978303 (PMC6466856; doi:10.1155/2019/6978303)
Supplement: Supplementary Materials — Figure S1: (A) schematic representation of donor template designing strategy. (B) Computer-generated sequencing electropherogram of Idua gene-corrected iPSC. Figure S2: phase contrast images of gene-corrected miPSCs: (A) before inducing differentiations; (B) 21 days after spontaneous differentiation induction; (C) 21 days after fibroblast differentiation induction. Scale bars: 100 μm. [file 6978303.f1.pdf]

Supplementary Figure 1

A

Mus musculus strain C57BL/6J chromosome 5
Idua gene (Ch5. 108,660,331~108,685,446)

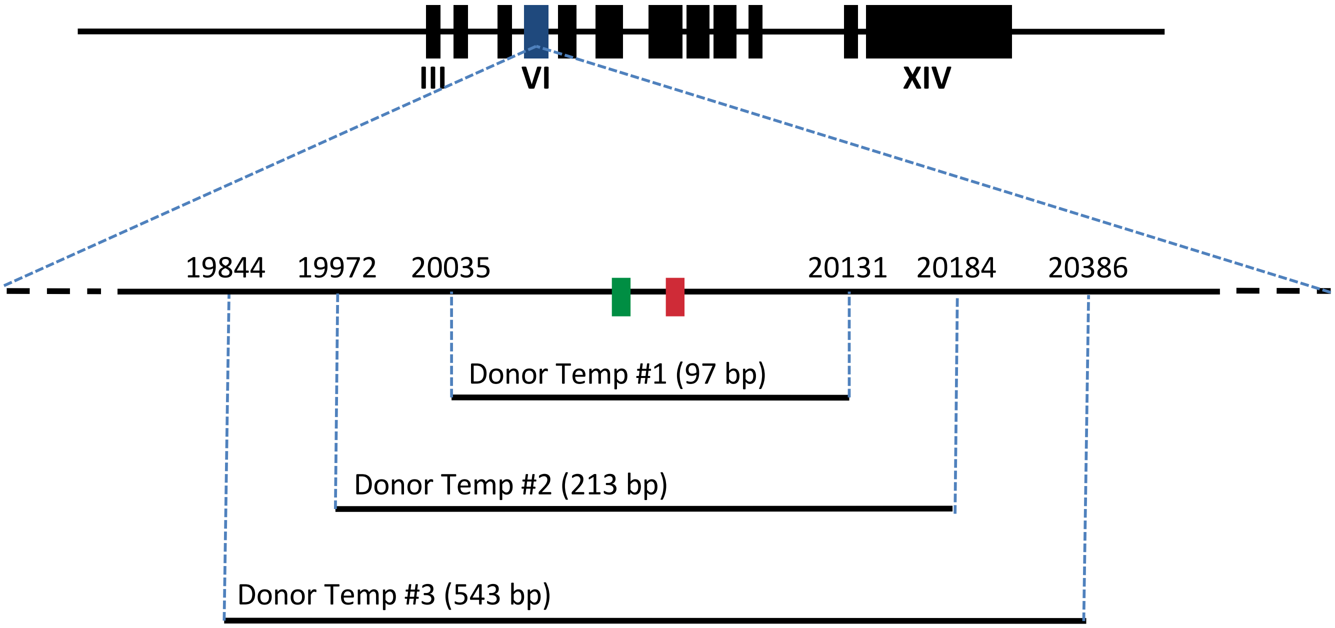

B

Post gene editing validation sequencing results

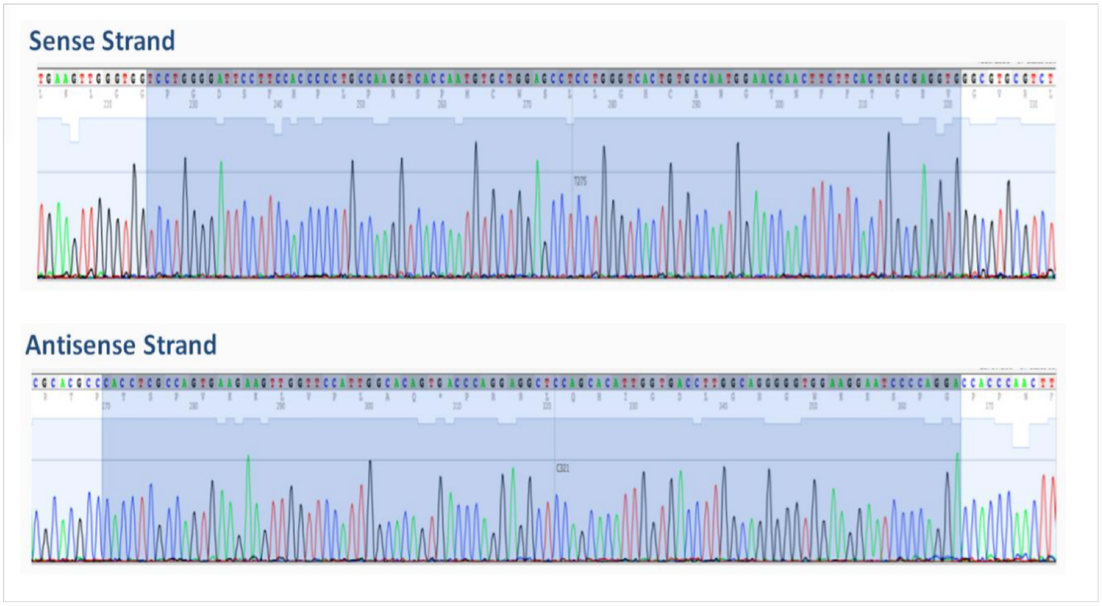

Supplementary Figure 2

A

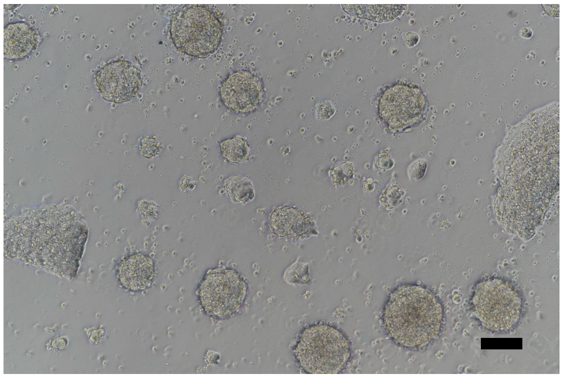

B

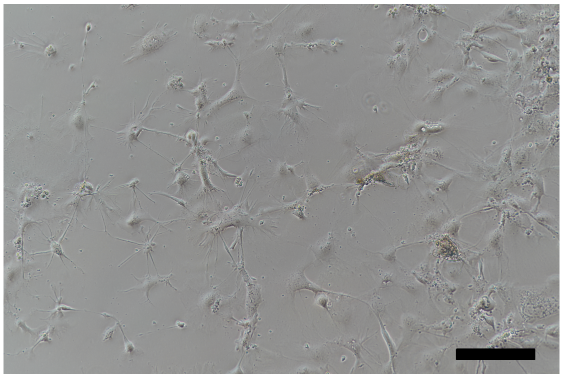

C

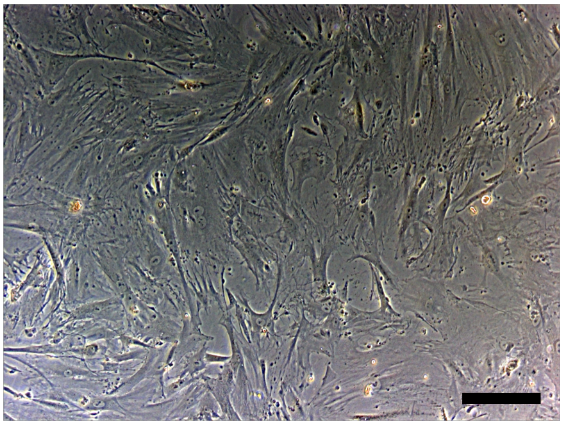

Supplementary Figure Legend

Figure S1. (A) Schematic representation of donor template designing strategy. (B) Computer-generated sequencing electropherogram of Idua gene corrected iPSC.

Figure S2.

Phase contrast images of gene-corrected miPSCs; (A) before inducing differentiations. (B) 21 days after spontaneous differentiation induction. (C) 21 days after fibroblast differentiation induction. Scale bars: 100 um

Supplementary Information

1. Target sequences for Guide RNAs:

1) ATTCCTTCCACCCCCTGCCAAGG (20043-20065)
2) CTCCTGGGTCACTGTGCCAATGG (20084-20106)
3) AGCCCCACTTTGAAGTTGGGTGG (20012-20034)
4) TGGCGAGGTGGGCGTGCGTCTGG (20122-2144)
5) GCGTTTTTCCAGACCCTGTTGGG (19884-19906)
6) TACCCCTATTTACAATGACGAGG (20324-20346)

2. Donor Templates:

Template #1: 97 bp

5'-TCCTGGGGATTCCTTCCACCCCCTGCCAAGATCACCAATGTGCTGGAGC
CTCCTGGGTCACTGTGCCAATGGAACCAACTTCTTCACTGGCGAGGTG-3' (20035-20131)

Template #2 213 bp

5'-GAATTACTATGATGCCTGCTCTGAGGGGCTGCGCATTGCCAGCCCCACTTTGAAGTT
GGGTGGTCCTGGGGATTCCTTCCACCCCCTGCCAAGGTCACCAATGTGCTGGAGCCT
CCTGGGTCACTGTGCCAATGGAACCAACTTCTTCACTGGCGAGGTGGGCGTGCGTCT
GGATTACATCTCCCTGCACAAGAAGGTGTGTGGTGCTCCCAC-3' (19972-20184)

Template #3: 543 bp

5'-ATGACTTTGACAACGTGTCCATGACCACACAAGGTAGGCAGCGTTTTTCCAGACCCT
GTTGGGTGGGCTGTATGAGAGGGAAGCAGGACAATCCCAGACCCTCAAGACCCTCC
TGCCCTAGGCTTCCTGAATTACTATGATGCCTGCTCTGAGGGGCTGCGCATTGCCAG
CCCCACTTTGAAGTTGGGTGGTCCTGGGGATTCCTTCCACCCCCTGCCAAGGTCACC
AATGTGCTGGAGCCTCCTGGGTCACTGTGCCAATGGAACCAACTTCTTCACTGGCGA
GGTGGGCGTGCGTCTGGATTACATCTCCCTGCACAAGAAGGTGTGTGGTGCTCCCAC
CCCTCCCTGCCTCCTTTGAGCTGAGCCTGTACTGAGTGAGGTATGTGCTGGCTCAGG
GTGCAGGTAGCTCCATCGCCATCCTGGAGCAGGAGATGGCAGTTGTGGAGCAGGTC
CAGCAGCTCTTCCCTGAGTTCAAGGATACCCCTATTTACAATGACGAGGCAGACCCT
CTGGTGGGCTGGTCCCTGCCACAACCTTGGAG-3' (19844-20386)

3. Validation qPCR primers:

Neo^{r} primers:

Forward 5'-ATGGCTTTCTTGCCGCCA-3'

Reverse 5'-GAACCTGCGTGCAATCCATC-3'

IDUA primers:

Forward 5'-AGGTCACCAATGTGCTGGA-3'

Reverse 5'-CCTTCTTGTGCAGGGAGATG-3'

Sequencing primers:

Forward 5'-TAGGTATGGGCTGACACACG-3'

Reverse 5'-AAGGACCCACCTTCACCAC-3'

4. Mouse Idua gene target sequence:

TGCGCATTGCCAGCCCCACTTTGAAGTTGGGTGGTCCTGGGGATTCCTTCCACCCCCTGCCAAGGTCACCAATGTGCTGGAGCCTCCTGGGTCACTGTGCCAATGGAACCAACTTCTTCACTGGCGAGGTGGGCGTGCGTCTGGATTACA

Yellow and green highlighted sequences are targets of gRNA#1 and #2, respectively. The underlined sequence is the donor template sequence #1. The "G" in red font is in the native mouse Idua gene sequence.
